# Toll-Like Receptor-Based Strategies for Cancer Immunotherapy

**DOI:** 10.1155/2021/9912188

**Published:** 2021-05-22

**Authors:** Saghar Pahlavanneshan, Ali Sayadmanesh, Hamidreza Ebrahimiyan, Mohsen Basiri

**Affiliations:** ^1^Functional Neurosurgery Research Center, Shohada Tajrish Comprehensive Neurosurgical Center of Excellence, Shahid Beheshti University of Medical Sciences, Tehran, Iran; ^2^Department of Stem Cells and Developmental Biology, Cell Science Research Center, Royan Institute for Stem Cell Biology and Technology, ACECR, Tehran, Iran

## Abstract

Toll-like receptors (TLRs) are expressed and play multiple functional roles in a variety of immune cell types involved in tumor immunity. There are plenty of data on the pharmacological targeting of TLR signaling using agonist molecules that boost the antitumor immune response. A recent body of research has also demonstrated promising strategies for improving the cell-based immunotherapy methods by inducing TLR signaling. These strategies include systemic administration of TLR antagonist along with immune cell transfer and also genetic engineering of the immune cells using TLR signaling components to improve the function of genetically engineered immune cells such as chimeric antigen receptor-modified T cells. Here, we explore the current status of the cancer immunotherapy approaches based on manipulation of TLR signaling to provide a perspective of the underlying rationales and potential clinical applications. Altogether, reviewed publications suggest that TLRs make a potential target for the immunotherapy of cancer.

## 1. Introduction

Cancer immunotherapies have been of great promise for the treatment of different types of cancer. Checkpoint inhibitors such as anti-PD-1/PDL-1 and anti-CTLA4 antibodies that can treat solid cancers through activation of an antitumor immune response are now approved by regulatory organizations in Europe and the US [1]. Cell-based immunotherapies such as chimeric antigen receptor (CAR) T cells have also shown to be strikingly effective in treating refractory or relapsed hematopoietic malignancies [2]. These successful experiences showed that triggering an efficient antitumor immune response could be applied as an effective therapy for cancer. To this end, exploiting relevant immunostimulatory mechanisms is of great importance for the development of new potent immunotherapy strategies. Toll-like receptors (TLRs) are a class of molecules that play such immunostimulatory roles in many immune cells involved in cancer immunity.

TLRs are a well-known family of pattern recognition receptors that recognize conserved structures in pathogens. The extracellular TLR groups (TLR1, TLR2, TLR4, TLR5, TLR6, and TLR10) are expressed on the plasma membrane while intracellular TLR groups (TLR3, TLR7, TLR8, and TLR9) are expressed in the endosome and endoplasmic reticulum, whereas TLR4 is found both on the plasma membrane and in the intracellular compartments. These molecules detect infection-derived ligands through their extracellular/luminar domain known as leucine-rich repeats (LRRs) and signal through their cytosolic conserved region known as toll-like/interleukin-1 receptor (TIR) homology domain to trigger the downstream signaling adaptor proteins such as myeloid differentiation primary response gene 88 (MyD88) [3].

TLRs are expressed on a variety of cells, including innate immune system cells such as macrophages, neutrophils, dendritic cells (DCs), natural killer (NK) cells, and mast cells, as well as the adaptive immune system (T and B lymphocytes), stromal cells, and different types of tumor cells [4]. The wide expression and functional role of TLRs in the tumor-infiltrating immune cells and the intrinsic role of these cell types in cancer progression or anticancer immune response highlight the potential of TLR as a promising target for cancer immunotherapy. Therefore, pharmacological compounds that can activate TLR molecules have been extensively studied for boosting the immune response against malignant cells. As we discuss in the next sections, this strategy not only activates intrinsic immune cells but also can benefit adoptively transferred immune cells in cell-based immunotherapy settings. Moreover, since TLRs are potent stimulatory molecules in many immune cells, their signaling domains can be applied for engineering synthetic molecules such as CARs for genetic modification of therapeutic immune cells. In this review, we first discuss the role of TLRs in the immune microenvironment of cancer to provide a background on the roles played by TLRs in the cancer microenvironment. Then, we will survey current reports on the application of TLR modulators in cancer immunotherapy and the TLR-based strategies for boosting immune cell therapies of cancer.

## 2. TLRs in Cancer Immunity

### 2.1. Dendritic Cells

Although the frequency of DCs in the tumor microenvironment is low, they are very important to orchestrate antitumor response in the tumor microenvironment [5]. It is demonstrated that maturation of murine DCs directed by TLR stimulation primes antigen presentation and is pivotal for induction of T cell cytotoxicity. Signaling via activated TLR3 and TLR7 exerts maturation of DC subpopulations and enhances DC-directed immunogenicity mainly via IL-27-mediated signaling [6, 7]. Notably, activation of TLR3 on human breast cancer-associated dendritic cells has been shown to increase IFN-*λ* production which in turn directed IL-12 release. This IFN-directed phenotype provided a Th1 microenvironment and enhanced cytotoxic T cell activation [8]. Activation of TLR4 has been shown to enhance DC maturation which promoted anticolorectal cancer T cell response in vitro [9]. Similarly, stimulation of DCs via TLR-4 activation and tumor antigens significantly increased cytotoxic CD8+IFN*γ*+ T cells *in vivo* [10]. Stimulation of TLR7/8 on dendritic cells isolated from leukemic blasts of AML patients has been shown to promote efficient maturation and subsequently activate autologous cytotoxic T cells in vitro. Furthermore, targeting TLR7/8 was a crucial addition to TLR3, 2, or 4 activations to prime DC maturation and production of IL-12 [11].

Moreover, activation of plasmacytoid dendritic cells via TLR7 signaling interestingly resulted in murine melanoma cell killing. TLR7 enabled tumor-associated effector pDCs to educate NK cells, mDCs, as well as activation of CD8+ T cells [12]. A similar trend was also observed on ALL patients. pDC activation via TLR9 molecules directed IFN production which in turn activated NK cells by engagement of TRAIL and CD69 expression [13]. This antitumor TLR7 effect was also shown in CNS tumors which increased DC maturation and tumor-specific CD8+ T cells in mice [14]. Activation of DCs via TLR3 and TLR7/8 activated CD8+ T cell response and improved therapeutic activity of DC-targeted vaccines [15].

### 2.2. Macrophages

Macrophages are important immune cells functioning as tumor-protective subtype M1 or tumor-promoting M2 subtypes. It is well evident that skewing M2 subtypes in the tumor microenvironment to M1 can enhance antitumor properties of M1 macrophages [16]. Studies demonstrated the role of TLR3 activation to revert M2 phenotype to M1 mainly via recruiting IFN signaling cascade in vitro and in vivo. The latter resulted in the expression of costimulatory molecules such as CD80, CD86, and CD40 as well as immunostimulatory cytokines IL-6, IL-12, and TNF-*α*. Consequently, this resulted in improved antigen uptake by macrophages and become capable of activating T cells which augmented immune control of tumor growth in mice [17]. A similar result was obtained against Lewis lung carcinoma cancer cells in mice via TLR3 activation as well as TLR4 engagement in sarcoma-bearing mice [18, 19]. It has shown that TLR4 may have effects on macrophage migration via NF-*κ*B, TNF-*α*, and VEGF expression [20]. In another study, antitumor crosstalk between macrophages and NK cells via engagement of TLRs was shown ex vivo. This TLR-directed M1 antitumor phenotype was accompanied by the production of immunostimulatory cytokines such as IL-18 from human ovarian tumors and stimulated resting NK cells to produce IFN*γ* and Th1-type immune responses [21].

Furthermore, it is well documented that myeloid-derived suppressor cell (MDSC) differentiation to either M1 or M2 macrophages is possible in tumor environment which can exert antitumor or protumor effects, respectively [22, 23]. TLR7/8 activation has been shown to differentiate MDSCs in the tumor microenvironment towards M1 phenotype and enhance tumor regression in mice. This TLR-directed antitumor activity synergistically decreased oxaliplatin resistance in mice harboring colorectal cancer [24]. The role of TLR2/6 stimulation on macrophages to derive NK cell activation and T cell cytotoxicity was also reported in several tumors such as pancreatic cancer as well as metastasis mice models [25, 26]. This NF-*κ*B-directed signaling increases COX-2 expression on macrophages and monocytes to derive immune surveillance in tumors [25].

### 2.3. Tumor Cells

TLR8-mediated signaling in tumor cells reversed immune suppression in the tumor microenvironment via blocking cAMP production. This helped overcome naïve/effector T cell senescence in the immunosuppressive microenvironment of tumor cells [27]. Crosstalk between tumor cells and *γδ* T cells via TLR2 and TLR7 signaling has been reported. TLR2 and 7 activations directed CD54 expression in pancreatic adenocarcinoma and lung and head and neck carcinomas which significantly directed effector function of T cells in vitro [28]. TLR7 activation of tumor cell lines such as Hela S3, keratinocytes, and fibroblasts directly promoted tumor cell apoptosis [29]. Moreover, this antiapoptosis effect TLR7 activation can be directed via infiltrated cytotoxic lymphocytes (CTL), NK, and DCs in the tumor microenvironment. Activation of TLR3-promoted apoptosis of prostate cancer cells via PKC-alpha-dependent signaling in combinational therapy with 5-FU significantly increased apoptosis of human colon cancer cells [30, 31]. The direct antitumor effect of TLR5 activation has been reported in mouse xenografts of human breast cancer cells [32]. The same autocrine effect of TLR5 activation was also reported on glioma cells via NF-kappaB activation and NO production [33].

### 2.4. B Cells

B cells express a variety of TLRs which can transmit strong activation signals that synergize with B cell receptor signaling [34]. The role of TLR7, as well as TLR8, on enhancing B cell antibody and cytokine production is well documented. These activated B cells which are similar to CD40-activated B cells showed increased survival and upregulation of B7 costimulatory molecules [35]. Stimulation of TLR1/2, TLR7, and TLR9 in B cells induces the secretion of a wide range of cytokines and chemokines [36]. Increased expression of costimulatory molecules, enhanced cytokine production, and more efficient antigen presentation by B cells can consequently result in better activation of the helper T cells [34]. There are also several reports demonstrating that TLR stimulation promotes effector functions of B cells including proliferation, antibody production, and Ig class switching [37–39].

### 2.5. NK Cells

NK cells are a type of lymphocyte that functions as a first-line defense against tumor cells. It is well documented that almost all TLRs can be expressed on NK cells depending on the NK cell population. Among all, signaling via TLR3, 7, 8, and 9 had been shown as a crucial route in tumor biology. Activation of TLR3 which is highly expressed on human NK cell lines such as NK92, YTC12, and YTS resulted in enhanced cytotoxicity effects on K562 cells. Moreover, targeting TLR3 enabled NK cells to kill head and neck squamous cell carcinoma (HNSCC) by secreting IFN*γ* [40, 41]. Activation of nucleic acid-sensing TLRs such as TLR7, 8, and 9 has been reported to enable the antitumor activity of NK cells. Activation of these TLRs is highly dependent on other cells in the tumor microenvironment. Although the expression of TLR7/8 on NK cells is controversial [42, 43], some reports showed that stimulation of TLR7/8 expression on NK cells via cytokines released from other cells activates NK cells and promotes their proliferation. Secretion of IFN*γ* and IL-12 and other inflammatory cytokines via TLR7/8 stimulation enabled NK cells to kill HNSCC and melanoma B16 tumor cells while TLR9 triggered cytotoxic activity of these cells on melanoma cells [44–46]. It was also reported that activation of NK cells via TLR2 could accelerate the antitumor activity of HER2-targeted monoclonal antibody therapy *in vitro* and *in vivo* [47].

### 2.6. Effector T Cells

Certain TLRs are expressed on different types of T cells which can directly modulate T cell function and antitumor activity of these cells. TLR1/2, 5, and 7/8 activation has been reported to stimulate proliferation and cytokine production of memory CD4+ T cells. For instance, increased secretion of IFN*γ* and a slight increase in IL6 from freshly isolated *γδ* T cells have been reported via TLR2 and TLR3 stimulations. A high concentration of TLR5 ligand increased CD4+ T cell proliferation and IL2 expression [48]. Activation of several TLRs such as TLR2, 3, and 9 in purified B6 CD4+ T cells can act as a costimulatory signal for TCR activation [49]. TLR9 activation via NF-*κ*B signaling inhibits apoptosis in CD4+ T cells; similarly, signaling via activation of TLR2 stimulated CD8+ T cell survival [50]. TLR7 and TLR8 activation of CD4+ T cells helped proliferation and enhanced production of IFN*γ*, IL-2, and IL-10 [51]. Moreover, activation of TLR7 induced effector activity of CD8+ T cells via the MyD88 and AKT-mTOR pathway which is strongly dependent on glucose uptake *in vitro* [52]. Although as mentioned earlier, activation of DCs, NK cells, and Tregs can modulate CD8+ T cell function, it is well documented that different TLRs can directly modulate different properties of CD8+ T cells in the tumor microenvironment. TLR1/2 activation is believed to entail effector activity to CD8+ T cells. Granzyme B, perforin, TNF-*α*, and IFN*γ* production is augmented via TLR1/2 activation both in vivo and in vitro. Ligation of TLR1/2 on CTLs could generate antitumor activity against B16 melanoma cells and result in significant tumor reduction [53]. This enhanced CTL cytotoxicity is suggested to be at least partly via the mTOR pathway. Inhibition of mTOR, Akt, and PKC in T cells hindered cytotoxic activity of CD8 T cells dramatically [54]. It is reported that TLR3 activation can also modulate effector CD8+ T cell function and increase IFN*γ* production as a functional coreceptor [55]. Moreover, direct activation of CD8+ T cells via engaging TLR3 is reported in an in vitro assay using transgenic OT-1 (CD8+) T cells. This antigen-independent stimulation of CD8+ T cells was followed by robust expansion and increased expression of activation markers in vivo [56].

### 2.7. Regulatory T Cells

It is well documented that specific TLRs can modulate the suppressive activity of murine and human regulatory T cells (Tregs). Activation of TLR4 on Treg improved their survival and enhanced their suppressive activity. Besides, TLR5 activation in a low concentration of ligand increased Foxp3 expression and slightly enhanced suppressive activity of human CD4+CD25+ Treg [48]. In contrast, TLR8 activation of Tregs via TLR8-MyD88-IRAK4 signaling could significantly revert the suppressive function of these cells in a tumor-bearing mouse model [57]. The effect of TLR2 activation to promote Treg proliferation was shown in several experiments although its effect on reverting suppressive activity of these cells is controversial [58, 59]. For example, TLR2 activation was reported to directly promote proliferation of Tregs *in vivo*; however, it inhibits immunosuppressive activity [60]. Since the overall effects of most TLR agonists are towards the enhancement of antitumor effects (see next section), one can speculate that TLR-mediated stimulation of Tregs is not a major challenge for TLR-based immunotherapy. However, more precise investigations are required to address the underlying mechanisms that orchestrate immune cell interactions during systemic TLR stimulation.

## 3. TLR Agonists for Cancer Immunotherapy

Given the current knowledge on TLR and their ligands, various types of TLR agonists are developed including natural microbial components or synthetic ones and being used in anticancer therapy. These agents are considered immune-stimulating factors which enhance TLR signaling and activate an innate immune response that results in long-lasting adaptive immunity. The TLR agonists have been used for a variety of clinical applications. These agents could be used as vaccine adjuvants which caused strong TH1 and CTL response [61]. The MPLA is a TLR agonist that enhances immunity in hepatitis B vaccines [62]. The CpG ODN is also used in various vaccines such as hepatitis B, hepatitis C, and influenza which unlike complete Freund's adjuvant does not initiate local inflammatory reaction [63]. Another application of TLR agonist is for the treatment of allergic diseases with TLR agonists that enhance TH1 response, inhibiting TH2 development and related cytokines such as IL-4, IL-5, and IL-10 [64]. VTX-1463 (a TLR4 agonist) could reduce clinical symptoms in ragweed-allergic patients [65]. Treatment of infectious diseases is another application of TLR agonists. These molecules could enhance the specific immune response against microbial infections.

TLR agonists have been extensively studied for the enhancement of the immune response against cancer. These molecules could stimulate cytotoxic lymphocytes, natural killer cells (NK cells), and dendritic cells (DCs) which could be important characteristics in cancer therapy either in monotherapy or combined modal strategies. These agents are also used as vaccine adjuvants in humans to increase the immune response [66]. Although TLR agonists are generally considered potential anticancer agents, it should be noticed that the efficacy of some TLR antagonists may be dependent on the cancer type and the context of the immune system [67].

There are several examples of TLR agonists studied for boosting anticancer immune response. TLR2 agonists Pam3Cys (synthetic triacylated lipoproteins), SMP-105 (cell wall skeleton components), have been used for bladder cancer [68]. TLR3 stimulator poly I:C (a synthetic analog of viral dsRNA) increased production of type I IFNs and inhibited tumor cell proliferation [69], and ARNAX (DNA-capped dsRNA modulator, TLR3 agonist) increased CTL and memory cell numbers [70]. Multiple TLR4 activators have been studied in experimental and clinical trials, including AS04 (FDA-approved for cervical cancer), MPLA (derivative of lipid A, cervical cancer), and GLA-SE (G100-synthetic GLA, lymphoma tumor) [71, 72]. CBLB502 (natural flagellin/entolimod) and M-VM3 (Mobilan, a recombinant nonreplicating adenovirus encoding flagellin) are some of the TLR5 agonists which have been studied for head and neck cancer and prostate cancer, respectively [73, 74]. Imiquimod (a TLR7 agonist) is used for cervical, vaginal, and vulvar intraepithelial cancers [75]. Some of the important examples of TLR9 agonists are CpG-7909 (single-stranded CpG ODN, non-Hodgkin's lymphoma, renal cell carcinoma, melanoma, cutaneous T cell lymphoma and glioblastoma, and non-small-cell lung cancer), IMO2055 (CpG ODN-based oligonucleotide, advanced NSCLC), MGN1703 (natural DNA molecule, advanced solid tumors, small cell lung cancer), dSLIM (two single-stranded loops connected with double-stranded stem, metastatic colorectal cancer), SD-101 (follicular lymphoma), KSK-CpG (phosphorothioated CpG ODN, melanoma), ODN M362 (hepatocarcinoma), and CpG-1826 (enhance anticancer effect in glioma xenograft model) [68, 76–90].

**Figure 1:**
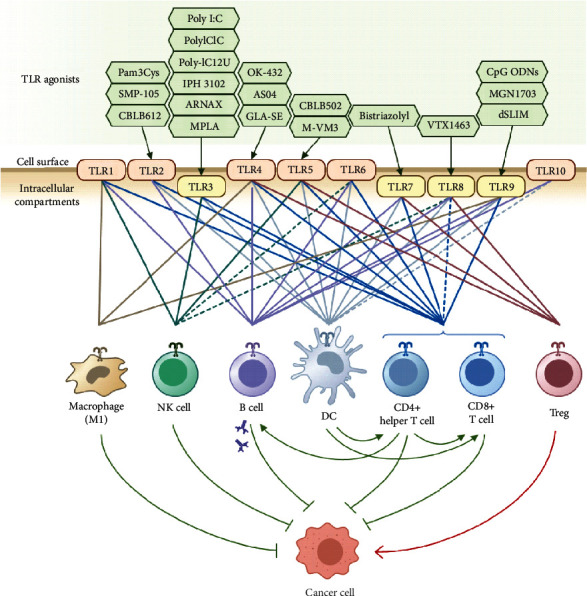
Targeting TLRs expressed on the immune cells in the tumor microenvironment with TLR agonists. Examples of TLR agonists are shown at the top. TLRs which are expressed in human cell surface or intracellular compartments are shown in the middle. The expression of each TLR on different immune cell types is indicated by color-coded lines. Some reported low-level expressions of TLRs with unknown functional status are ignored in this figure.

A list of TLR agonists which are mentioned above is summarized in Table 1 (see also Figure 1). Although there may be more established or candidate TLR agonists under different phases of preclinical and clinical studies, they follow more or less similar biological strategies to induce TLR signaling. Another strategy is to combine TLR agonists with other immunotherapy agents to obtain a synergistic effect. For instance, combining TLR7 and TLR9 agonists (1V270 and SD-101) with anti-PD1 checkpoint inhibitor activated tumor-infiltrated macrophages and induced a potent antitumor immune response, preventing primary tumor growth and metastasis in a mouse model of head and neck cancer [91]. Similar enhancement in the immune response was obtained by combining the agonists of TLR9 (ODN1826 or MGN1703) with CTLA-4 or PD-1 blockade in a mouse model of poorly immunogenic melanoma [92]. It is noteworthy that some TLR antagonists including small molecules, interfering RNAs, and antibodies also have been used for cancer therapy. However, the rationale behind the application of these TLR inhibitors is to target protumor TLRs expressed on the malignant cells and not the immune cells. One of the challenges with these antagonists is that they cannot target TLRs specifically on the tumor cells and may cause unfavorable effects on the immune microenvironment of cancer. We do not cover this approach here since it is not considered immunotherapy; thus, it is out of the scope of this review and has been reviewed elsewhere [93].

## 4. TLR-Based Strategies in Immune Cell-Based Therapy of Cancer

Several types of immune cells including T cells, NK cells, DCs, and macrophages have been used for cancer immunotherapy, as reviewed elsewhere [116–119]. Since TLRs are involved in the regulation of these immune cells, it is plausible that manipulation of TLRs modifies and improves these cell-based immunotherapy methods. To exploit TLR-based regulation for enhancing transferred cell-based immunotherapy, two general strategies are conceivable: first, to utilize TLR or their derivative domains for genetic modification of the immune cells to augment their functionality against the malignant cells; second, to administrate a TLR agonist or antagonist along with the immune cells to provide them with inflammatory and costimulatory signals. In this section, we will discuss current progress in these approaches and possible future opportunities.

**Figure 2:**
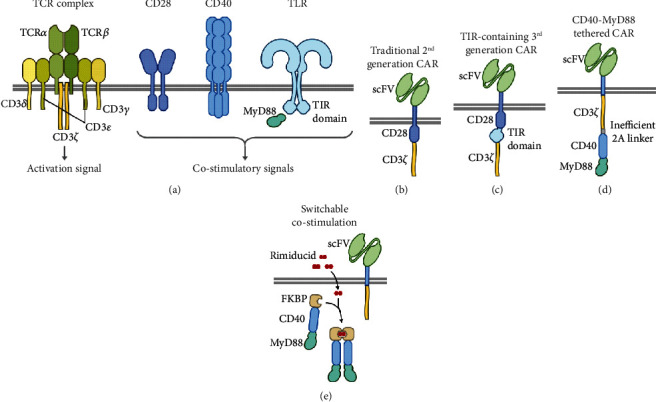
TLR-based strategies for improvement of CAR T cells. (a) Schematic presentation of wild-type T cell proteins from which signaling domains are derived to construct chimeric synthetic receptors. (b) Traditional second-generation CAR containing an scFV domain for antigen recognition on the extracellular portion and CD3*ζ* and costimulatory signaling domains on the cytosolic side (CD28 domain is shown here as an example). (c) A third-generation CAR with a TIR signaling domain derived from TLRs. (d) A CD40-MyD88 fusion protein tethered to a first-generation CAR through an inefficient 2A linker. (e) The CD40-MyD88 fusion is linked to a rimiducid-binding domain from an FKBP protein to form a pharmacological switch that can transmit a costimulatory signal by dimerization upon rimiducid treatment.

Genetic modification of immune cells provides a valuable means for directing their function towards the tumor cells and arms them with transgenes that confer resistance against the harsh immunosuppressive microenvironment of the tumor. For instance, genetic modification of T cells by CARs enables them to recognize and kill the tumor cells via surface antigens [120]. CAR molecule is an engineered transmembrane protein that can recognize a specific antigen by its extracellular domain, typically a single-chain fragment variable (scFV), and transmit activation signals through its cytosolic domains [121]. Signals transmitted by the cytosolic domains play a crucial role in regulating different aspects of cytotoxicity, memory formation, and persistence of CAR T cells [122]. First-generation CARs harboring only a CD3*ζ* signaling domain proved inferior to the second-generation CARs which contain an additional signaling domain from a costimulatory receptor such as CD28 (Figures 2(a) and 2(b)) [123]. Since TLRs provide potent costimulatory signals for T cell activation, their intracellular domains also can be used for the construction of the CAR molecules. Accordingly, a third-generation anti-CD19 CAR containing CD28, CD3*ζ*, and TLR2 signaling domains (1928zT2) revealed the synergistic effect between TLR2 and CD28 costimulatory signals (Figure 2(c)) [124]. A recent study shows that tethering a TLR adaptor molecule, MyD88, along with a CD40 signaling domain to the cytosolic domain of a first-generation CAR construct promotes CAR T cell survival, proliferation, and antitumor activity (Figure 2(d)) [125]. The same MyD88-CD40 fusion protein when linked to a rimiducid-binding domain (FKBP12v36) forms an inducible switch molecule that can enhance *in vivo* expansion and persistence of CAR T cells upon systemic administration of the small molecule rimiducid (Figure 2(e)) [126]. Previous studies have shown that signaling through different costimulatory domains results in distinct functional characteristics in CAR T cells in terms of memory differentiation, persistence, and toxic side effects [122, 127], implying that the selection of the costimulatory domain can be applied to fine-tune CAR T cell function. Therefore, the aforementioned TLR-derived costimulatory domains can expand the signaling domain arsenal to further customize CAR architecture and function.

Besides T cells, TLR signaling components have also been used in the context of other immune cells that can also be genetically engineered by specific variants of CARs. For instance, the aforementioned rimiducid-inducible molecular switch composed of MyD88, CD40, and FKBP12v36 domains (Figure 2(e)) has been used in NK cells genetically modified with CAR and IL-15, resulting in the robust proliferation and prolonged persistence in vivo [128]. Another example of the application of TLR-derived domains for genetic engineering of the immune cells comes from CAR-modified macrophages. Macrophages genetically modified to express CAR molecule consisting of an extracellular scFV domain and a cytosolic TIR domain are reported to show antigen-specific cytotoxicity and expansion both *in vitro* and *in vivo* [129, 130]. These instances suggest that genetically engineered receptors mimicking TLR stimulation may be used to modify immune cells which naturally express and get stimulated by TLRs.

As mentioned earlier, agonists of different TLRs can trigger the antitumor response by modulating specific types of endogenous immune cells. The same principle can be applied for upregulating the adoptively transferred immune cells. For instance, poly I:C (a TLR3 ligand) treatment has been shown to synergize with an antiepidermal growth factor receptor variant III (EGFRvIII) CAR T cell therapy in immunocompetent xenograft models of colon and breast cancer [131]. In this setting, poly I:C mediated its effect through type I IFNs and downregulation of immunosuppressive myeloid-derived suppressor cells (MDSCs). TLR agonists have also been used for ex vivo activation of the immune cells before being transferred for cancer therapy. For example, coculture of NK cells and DCs in the presence of lipopolysaccharide, which is TLR4 agonist, resulted in superior DC maturation with potent antitumor activity when transferred to a mouse colon cancer model [132]. This approach can be extended to other immune cell therapies and various TLR agonists and antagonists, considering the compelling data on the effect of the TLR modulators on the antitumor effects of T cells [133], NK cells [134], DCs [135], and macrophages [136].

## 5. Recent Clinical Trials on TLR-Based Cancer Immunotherapy

TLR agonists have been under clinical investigation for years and in several cases showed potential therapeutic efficacy against cancers. The US Food and Drug Administration (FDA) has approved several TLR agonists for cancer treatment, such as BCG (a primarily TLR2/4 agonist) for bladder noninvasive transitional cell carcinoma, AS04 (a TLR4 agonist) for cervical cancer, and imiquimod (a TLR7 agonist) for superficial basal cell carcinoma [137]. More recent published results on clinical investigation of TLR agonists in cancer (Table 2) showed the benefit of these molecules when combined with another immunotherapy agent. For example, combining peptide or recombinant cancer vaccines with agonists of TLR3, 4, and 9 showed improved immune stimulation and enhanced T cell response in melanoma patients [99, 138, 139]. The addition of TLR8 agonist Motolimod (VTX-2337) improved the cellular antitumor immune response induced by an anti-EGFR antibody (cetuximab) in head and neck squamous cell carcinoma [140, 141]. One report described the simultaneous administration of cell-based immunotherapy with a TLR3 agonist, where treatment with peptide-pulsed DCs plus poly-ICLC was tolerated and induced a detectable tumor-specific T cell response in patients with pancreatic cancer [142]. However, the addition of TLR2 agonist CADI-05 to chemotherapy with cisplatin-paclitaxel did not provide any survival benefit for non-small-cell lung cancer patients in a phase II clinical trial [143]. Another study that combined paclitaxel chemotherapy with a TLR7 agonist (imiquimod) showed a short-lived improvement in disease regression [144].

Trends of the ongoing clinical trials also suggest that combinatorial strategies are being pursued in the field of TLR agonists. A summary of ongoing clinical trials started between 2019 and April 2021 is provided in Table 3. Clinical trials started before this period are reviewed elsewhere [137]. Most of these recently started studies are on a combination of TLR inhibitors with another immunotherapy agent including PD-1 blockers, CTLA-4 blockers, and agonistic anti-OX40 antibodies. This is in line with recent preclinical findings that also suggest that this kind of combination may produce a synergistic effect to evoke a more effective immune response [91, 92].

## 6. Conclusion

TLRs are present on multiple immune cells within the tumor microenvironment. Given the important role of these receptors on the regulation of immune response, they are considered to be promising targets for modulation of the immune system towards a potent antitumor response. Recent data on the application of TLR agonists in experimental and clinical settings demonstrate the potential of this strategy for cancer treatment. Although current studies have shown the proof of concept for the application of TLR-targeted drugs for cancer treatment, given the variations in tumor immunophenotypes, it is likely that cancer type and microenvironment condition among other factors may affect the clinical outcome of TLR-targeting immunotherapies. These differences need to be addressed especially when preclinical animal experiments are conducted. More recent data suggest that combining TLR antagonists with other immunotherapy approaches, such as checkpoint inhibitors and cell-based immunotherapy, could boost the efficacy of immunotherapy regimens [91, 131]. The trend of recent clinical trials also suggests that combinatorial therapy containing a TLR agonist as a booster for immune response is considered as a more promising approach by the investigators (Tables 2 and 3). As discussed above, the application of TLR-based genetic engineering strategies may also provide new options for cancer immunotherapy. Since previous experiences with other costimulatory domains showed the impact of the domain architecture of engineered systems on the clinical outcome, it is necessary for future studies to make a head-to-head comparison between TLR-containing engineered receptors with other synthetic structures in preclinical and clinical settings to elucidate the characteristics of TLR-derived signals in the engineered immune cell therapies and its impact in the clinical outcome.

## Figures and Tables

**Table 1:** A summary of TLR agonists with their corresponding TLR targets.

Target TLR	Agonist	Molecule type	References
TLR2	Pam3Cys	Lipoproteins	[94]
	SMP-105	Cell wall skeleton components	[95, 96]
	CBLB612	Lipopeptide	[97]
TLR3	Poly I:C	Synthetic dsRNA	[98]
	Poly-ICLC		[99]
	Poly-IC12U		[100]
	IPH 3102		[101]
	ARNAX		[102, 103]
TLR4	MPLA	Lipid	[104]
	GLA-SE	Lipid	[105]
	AS04	MPL and aluminum hydroxide	[106]
	OK-432	Low virulence *Streptococcus pyogenes* strain	[107]
TLR5	CBLB502	Protein	[61]
	M-VM3	Adenoviral vector	[74]
TLR7	Bistriazolyl	Small molecule	[108]
TLR8	VTX1463	Small molecule	[109]
TLR9	MGN1703	Polynucleotide	[61]
	CpG-7909	Oligonucleotide	[110]
	IMO2055		[111]
	dSLIM		[61]
	SD-101		[112, 113]
	KSK-CpG		[88]
	ODN M362		[114]
	CpG-1826		[115]

**Table 2:** Clinical studies on the application of TLR agonists for cancer treatment published since 2016.

Target TLR	TLR agonist	Companion treatment	Conditions	Phase	Results	Reference
TLR2	CADI-05	Chemotherapy (cisplatin-paclitaxel)	Non-small-cell lung cancer	II	No survival benefit was observed with the addition of CADI-05 to chemotherapy.	[143]
TLR3	Poly-ICLC	NY-ESO-1 peptide vaccine	Melanoma	I/II	Enhanced specific CD8+ T cell response.	[138]
		Peptide-pulsed DCs	Pancreatic cancer	I	The treatment was safe and induced a measurable tumor-specific T cell population.	[142]
TLR3, TLR4	Poly-ICLC, LPS	Multipeptide vaccine and incomplete Freund's adjuvant	Melanoma	I	Combinations of poly-ICLC or LPS with peptide vaccine and incomplete Freund's adjuvant are safe and induce T cell response.	[99]
TLR4 TLR9	AS15	Recombinant MAGE-A3 vaccine	Melanoma	I	The treatment was tolerated and produced durable Ab responses.	[139]
TLR7	Imiquimod	Chemotherapy (paclitaxel)	Breast cancer cutaneous metastases	II	The combination was effective in inducing disease regression, but responses were short-lived.	[144]
TLR8	Motolimod	Anti-EGFR (cetuximab)	Head and neck squamous cell carcinoma	I	The addition of TLR agonist enhanced the cellular antitumor immune response.	[140, 141]
TLR9	GNKG168	N/A	Minimal residual disease positive acute leukemia	I	Immunologic changes were observed.	[145]

**Table 3:** A summary of recent ongoing clinical trials on TLR agonists started after 2019.

Target TLR	TLR agonist	Companion treatment	Conditions^∗^	Phase	Status	NCT number
TLR2/4	BCG, PPD, Typhim Vi	Chemotherapy, radiofrequency ablation	Colorectal cancer	I	Not yet recruiting	NCT04062721
TLR3	Poly-ICLC	Peptide vaccine, anti-CD40	Melanoma	I/II	Recruiting	NCT04364230
	Rintatolimod	Anti-PD-1, chemotherapy	Ovarian cancer recurrent	I/II	Recruiting	NCT03734692
TLR4	GLA-SE	N/A	Lymphoma, T cell, cutaneous	II	Withdrawn	NCT03742804
		Anti-CTLA-4, anti-PD-1, chemotherapy	Colorectal cancer metastatic	I	Withdrawn	NCT03982121
TLR7	BNT411	Anti-PDL-1, chemotherapy	Solid tumor, lung cancer	I/II	Recruiting	NCT04101357
	Imiquimod	Anti-PD-1, focused ultrasound ablation	Multiple solid tumors	I	Recruiting	NCT04116320
	RO7119929	Anti-IL-6 receptor	Biliary tract and liver cancer	I	Recruiting	NCT04338685
	SHR2150	Chemotherapy, anti-PD-1, anti-CD47	Solid tumor	I/II	Recruiting	NCT04588324
TLR7/8	TransCon	Anti-PD-1	Solid tumors	I/II	Recruiting	NCT04799054
TLR8	Motolimod	Anti-PD-1	Carcinoma, squamous cell	I	Recruiting	NCT03906526
TLR9	CMP-001	Anti-OX40	Pancreatic cancer, unresectable solid neoplasm	I/II	Not yet recruiting	NCT04387071
		Anti-PD-1	Melanoma	II	Recruiting	NCT04708418
						NCT04401995
	SD-101	Radiation therapy, anti-PD-1	Pancreatic cancer	I	Recruiting	NCT04050085
		Anti-OX40	Malignant solid neoplasm	I	Recruiting	NCT03831295
	Tilsotolimod	Anti-CTLA-4, anti-PD-1	Advanced cancer	I	Recruiting	NCT04270864

^∗^Indicated conditions are summarized in this table. Complete information is available via the provided ClinicalTrials.gov identifier (NCT number).
